# 3D Printing of Chitosan Scaffolds and Films with Varying Roughness for Cultivation of Human Retinal Progenitor Cells

**DOI:** 10.1021/acsomega.5c05018

**Published:** 2025-09-26

**Authors:** Amalie Solberg, Natalia Robles-Anda, Eva Pasquier, Helena Dorado Monreal, Miguel Ladero, M. Esther Gallardo, Gary Chinga-Carrasco

**Affiliations:** † RISE PFI, Høgskoleringen 6b, 7034 Trondheim, Norway; ‡ Translational Research with iPS Cells Group, 542148 Research Institute of Hospital 12 de Octubre, imas12, 28041 Madrid, Spain; § FQPIMA Group, Chemical Engineering and Materials Department, 16734 Universidad Complutense de Madrid, 28040 Madrid, Spain

## Abstract

Chitosan was used for three-dimensional (3D) printing of films and well-resolved scaffolds. Three different molecular weights with a comparable degree of de-*N*-acetylation were studied for 3D printing; inks were characterized using rheology, and the resulting two-dimensional (2D) and 3D architectures were characterized by scanning electron microscopy (SEM). Printing and fixation were optimized for retention of shape fidelity. The films and scaffolds were functionalized with a short peptide containing the integrin-binding arginylglycylaspartic acid (Arg–Gly–Asp, RGD) sequence using a postprinting grafting approach. The 2D films and 3D scaffolds prepared were studied as support materials for human retinal progenitor cells (hRPCs), and the properties of native and RGD-modified chitosan were compared as support materials for hRPCs. The adhesion and proliferation of hRPCs were studied over a period of 6 or 18 days, and Matrigel was used as a positive control. Grafting with the RGD-containing peptide generally improved the biocompatibility of the materials. When comparing films with varying surface roughness resulting from the method used for drying, the cellular response differed significantly. The best performing material was air-dried chitosan films, which resulted in the formation of axons and larger cell clusters with observable live cells after 18 days of culture time. This work demonstrates effective methods for the preparation of 3D printed architectures and the promise of these materials for cell therapies and bioengineering applications.

## Graphical abstract

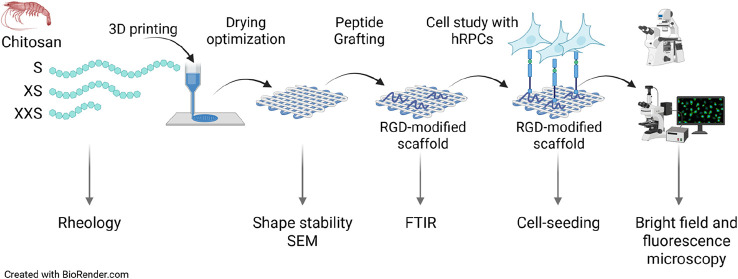

## Introduction

Engineering of biomaterials with tailored properties is important for the development of new stem-cell-based therapies. Stem cell treatment offers tremendous potential for several conditions, including degenerative retinal diseases like glaucoma.^1^ One bottleneck for clinical use of stem cell therapies is the scalability of the process.^2^ A strategy for the scaling of stem-cell-based treatments is the development of suitable scaffold materials allowing for the cultivation, attachment, spreading, and directed differentiation of human-induced pluripotent stem cells (hiPSCs).^3^ Biopolymers represent a diverse class of naturally occurring polymers with a range of physiochemical properties considered promising for the preparation of scaffolds.^4^ In nature, biopolymers form a range of hierarchical structures that facilitate adhesion and differentiation of cells in biological systems.^5,6^

Chitosan is a cationic biopolymer prepared from chitin. Chitin is the second most abundant polysaccharide on Earth, found predominately in the cell wall of yeast and fungi and in the exoskeleton of crustaceans.^7^ Chitin has a linear structure composed of β-1,4-linked *N*-acetyl-d-glucosamine (GlcNAc), and chitosan is prepared by partial or complete de-*N*-acetylation of chitin. The degree of de-*N*-acetylation (DDA) will strongly influence the properties of chitosan, including solubility,^8^ rheological properties,^9^ and bioactivity for some systems.^10^ Chitosan is defined as having a DDA of 50% or higher and is often described as the only naturally occurring polycation.^7^ The polyelectrolyte properties of chitosan make it particularly interesting for material engineering, taking advantage of its inherent pH-dependent solubility.^8^ Chitosan has been studied as a scaffolding material for multiple cell types, including for the slow proliferation of retinal stem cells with phenotype retention for use in transplants.^11^ However, despite the promise of using nonmodified chitosan, methods for the preparation of materials with a well-defined architecture from unmodified chitosan remain a challenge. While there are multiple examples of grafting strategies used for controlling the material properties of chitosan, such modifications most often take use of the amino group and inherently change the physiochemical properties.

Three-dimensional (3D) printing is an advanced fabrication technique that allows for the preparation of scaffolds with specific architectures, provided that the inks have good printability and fidelity of structures. For chitosan printing, the viscosity of the ink is influenced by the chain length distribution and DDA, in addition to the concentration, temperature, and deformation during extrusion.^12^ The nature of the ink and the resulting mechanical and structural features of the scaffold are critical for subsequent applications, such as support materials for cell cultures, where parameters like stiffness and porosity are of great importance for cellular attachment and proliferation.^13^ Understanding how the printability of chitosan can be optimized while retaining its inherent properties will allow for the preparation and evaluation of native chitosan for biomaterial applications.

In addition to mechanical characteristics, suitable surface chemistry is needed for the use of scaffolds as support materials for cell cultures. Introducing integrin-binding motifs such as arginylglycylaspartic acid (Arg–Gly–Asp, RGD)-containing peptides on the material surface is a common strategy for promoting cell adhesion and spreading.^14^ Previously, Arg–Gly–Asp–Serine (RGDS) peptides have been reported to result in the highest density of attached rat osteosarcoma cells, compared to Arg–Gly–Glu–Ser (RGES) motifs and pure chitosan.^15^ Increased cell spreading and proliferation have also been observed for RGD-grafted chitosan for human dental pulp stem cells (hDPSCs) and attributed to the introduction of RGD-adhesion motifs supporting cell anchoring, which is lacking in native chitosan.^16^ One much-used strategy for the grafting of chitosan with carboxyl acids involves taking advantage of the C-2 amino group using carbodiimide chemistry.^17^ Peptides can be grafted from their carboxyl termini, and an RGDS peptide, serine, will participate in the amide bond, while the remaining amino acids (RGD) will remain unperturbed.

Here, we show the preparation of (2D) films and (3D) scaffolds from native chitosan with different molecular weights (99, 201, and 340 kDa) and a comparable DDA. Rheology was used for characterization of the chitosan inks, and 3D printing was optimized for the preparation of scaffolds and films. The films and scaffolds could be functionalized with an RGDS peptide. Chitosan scaffolds and films having marked different surface characteristics, both native and functionalized with RGD peptides, were subsequently tested as support materials for human retinal progenitor cells (hRPCs). Biocompatibility was confirmed, and peptide grafting enhanced cellular attachment and proliferation. Air-dried chitosan films were the most promising for the formation of axons and larger clusters of cells. Taken together, chitosan is well suited for 3D printing of 2D films and 3D porous scaffolds, and with appropriate surface chemistry and structuring of the material, it shows strong promise as a support material for hRPCs.

## Materials and Methods

### Materials

Three chitosan samples were kindly supplied by Chitinor AS (Norway): chitosan XXS (weight-average molecular weight (*M*_w_) 99 kDa), chitosan XS (*M*_w_ 201 kDa), and chitosan S (*M*_w_ 340 kDa) (Table 1). All other chemicals were of analytical grade and purchased from Sigma.

**Table 1:** Chitosan Grades Supplied by Chitinor AS

code	commercial name	*M*_w_ (kDa)	DDA
chitosan XXS	chitopharm CM XXS	99	81
chitosan XS	chitopharm S	201	83
chitosan S	chitopharm S	340	85

### Rheological Assessment of Chitosan Gels

Chitosan gels were prepared by dissolving dry chitosan powder in an acetic acid (3 wt %) solution under continuous stirring at room temperature (RT) overnight. All rheological measurements were performed by using an MCR 702e rheometer. A 50 mm serrated cone plate geometry with a solvent trap was used with a gap of 0.20 mm. Strain sweeps were performed at 1 and 30 Hz and 20 °C, and frequency sweeps were performed below 20% strain at 20 °C.

### 3D Printing of Chitosan

The gels were printed with a Regemat3D bioprinter (BIO V1, Regemat 3D) upgraded with a freezing stage. The 3D printing settings are provided in Table 2.

**Table 2:** Parameters for 3D Printing of Chitosan Gels by Microextrusion

	chitosan XXS (99 kDa) (6 wt %)	chitosan XS (201 kDa) (6 wt %)	chitosan S (340 kDa) (6 wt %)
temperature for the syringe [°C]	RT	RT	RT
temperature for the bed [°C]	–15	–15	–15
nozzle diameter [μm]	250	250	250
head speed [mm/s]	1	2	1
flow speed [mm/s]	0.3	1.5	1
layer height [mm]	0.25	0.25	0.25
perimeter diameter [mm]	20	20	20
number of layers	2	2	2

In order to find the most adequate parameters for stabilizing the gels and drying them, different strategies were applied (Table 3). Drying optimization was done with chitosan XS because this grade provided a suitable shear thinning behavior for the 3D printing system applied in this study. Scaffold shrinkage after gelation and drying was quantified by scanning the scaffolds with an Epson Perfection scanner and measuring their size with image analysis (ImageJ).^18^

**Table 3:** Strategies for Stabilizing and Drying the 3D Printed Scaffolds Prepared with Chitosan XS (201 kDa, 83 DDA) in 3% AcOH^a^

sample	strategy for stabilization: gelation/washing/drying	gelation	washing	drying
1	EtOH/EtOH/air	1 M NaOH/ethanol, 24 h, 27 °C	ethanol RT	air drying
2	EtOH/water/freeze-dried	1 M NaOH/ethanol, 24 h, 27 °C	water RT	freeze-drying
3	EtOH/water/air	1 M NaOH/ethanol, 24 h, 27 °C	water RT	air drying
4	Water/water/freeze-dried	1 M NaOH/water, 1 h, RT	water RT	freeze-drying

a After gelation, the solvent (EtOH or water) was first used to remove excess NaOH before the washing step.

EtOH/water/freeze-dried was the strategy used for manufacturing 3D printed porous scaffolds and followed the procedure of Rogina et al.^19^ with some variations. The 3D printed chitosan samples were frozen in a freezer at −27 °C after printing. The samples were immersed in a gelation medium consisting of a final 1 M NaOH/ethanol (1:1 vol) solution at −27 °C for 24 h. After gelation, the samples were immersed in ethanol for 8 h at −27 °C for washing, followed by washing in water at RT for 16 h. The scaffolds were then freeze-dried.

### Film Preparation

Preliminary trials revealed that the high opacity of 3D printed porous scaffolds hindered the use of light microscopy to study the cell morphology and interaction with the material. Hence, chitosan films were used as model surfaces to evaluate the biocompatibility of chitosan with hRPCs. Chitosan films were prepared by 3D printing one layer of chitosan XS using similar printing parameters to those in Table 2. The difference is that the stage was not frozen but kept at room temperature. The films were printed as solid structures, i.e., without spaces between the 3D printed filaments. The filaments thus merged and formed a film of a uniform thickness. The films were then cross-linked in 1 M NaOH for 30 min at room temperature and washed with water. Half of the films were air-dried and half were freeze-dried to model the surface of 3D printed scaffolds.

### Peptide Grafting

Postprint grafting of the scaffolds was done using carbodiimide chemistry with an RGDS peptide (Arg–Gly–Asp–Ser) using the method reported by Taylor et al.^17^ In brief, EDC-HCl (3.12 mM) and NHS (3.48 mM) were dissolved in PBS (pH 7.4). The Arg–Gly–Asp–Ser peptide (RGDS peptide) (0.5 mM) was added, accounting for its solubility limit (25 mg/mL). The solution was placed on stirring, allowing all chemicals to dissolve (room temperature) before the chitosan scaffold was immersed in the solution. The reaction was left with gentle shaking at 25 °C for 20 h. After the reaction, the scaffold was rinsed first with PBS and then with MQ water (placed in a beaker with clean water for 2–3 min, repeated 3–4 times). Finally, the scaffolds were freeze-dried.

### Fourier Transform Infrared (FT-IR)

A PerkinElmer Spectrum 3 instrument was used for attenuated total reflectance (ATR) Fourier transform infrared (FT-IR) spectroscopy. ATR FT-IR spectroscopy was used to characterize the scaffold before and after peptide grafting by mounting and pressing the scaffolds between the prism and the torque arm. A total of 32 scans were used for all samples, and spectra were recorded in the region 500–4000 cm^–1^. The spectra were baseline-corrected using PerkinElmer Spectrum IR Software (version 10.7.2.1630) and normalized using the C–O–C absorption band at 1029 cm^–1^.

### Scanning Electron Microscopy (SEM) of 3D Printed Samples

Scaffolds were prepared for SEM analysis (SU3500, Hitachi) in secondary electron imaging (SEI) mode. The 2D films and 3D scaffolds were dried and sputtered with gold to make the samples conductive under the electron beam. The acceleration voltage during image acquisition was 5 kV.

### Generation and Human Retinal Progenitor Cell (hRPC) Culture

To perform the cell culture studies, hRPCs derived from a healthy human-induced pluripotent stem cell (hiPSC) line N44SV.1 previously created in our laboratory were used.^20^ hiPSC differentiation into hRPCs was carried out following the procedure reported by Lee et al.^21^ with minor modifications.^22^ For hRPC culture, N2B27 medium was employed (detailed composition is provided in Table 4).

**Table 4:** N2B27 Medium Composition for the Maintenance of hRPCs^21^

N2B27 medium			
composition	concentration	supplier	code
DMEM/F-12		Gibco	11320-033
N2 supplement (100×)	1×	Gibco	17502001
B27 supplement without vitamin A (50×)	1×	Gibco	12587-001
human FGF-2	20 ng/mL	Miltenyi Biotec	130-093-838

### Cell Seeding into Chitosan Films and Scaffolds

For seeding the hRPCs on the chitosan films and scaffolds, a method based on the Asghari Sana et al.^16^ protocol was followed. hRPC studies on the biomaterials were performed using nontreated 12-well culture plates (Corning, New York, NY; #351143). Before cell seeding, scaffolds were sterilized via wetting using 70% (v/v) ethanol for 1 h and washed three times with PBS 1×. Then, both sides were placed under UV light for 20 min. Chitosan films were also sterilized by using UV light. After film and scaffold sterilization, 0.45 × 10^6^ hRPCs in 60 μL of N2B27 medium were seeded into these samples and incubated at 37 °C and 5% CO_2_. Four hours later, 500 μL and 1 mL of cell culture medium were added to the films and scaffolds with the cells, respectively (day 0 of cell culture). The materials with the hRPCs were cultured for 6 days on the 3D printed scaffolds and 18 days on the 2D films, changing the culture medium every day. hRPCs cultured on plates coated with Matrigel Growth Factor Reduced (Corning, New York, NY; #354230) were used as a positive control for chitosan film experiments.

### Cell Attachment Verification by DAPI Staining

The hRPCs deposited on chitosan scaffolds were fixed with a 10% formalin solution (Merck, Darmstadt, Germany; no. HT501128) for 30 min at room temperature (RT). Then, cells were permeabilized using 0.1% Triton X-100 (Merck, Darmstadt, Germany; #T8787) in Tris-buffered saline (TBS) for 45 min at RT. Afterward, blocking was performed with 3% donkey serum (Merck, Darmstadt, Germany; #D9663) and 0.3% Triton X-100 in TBS for 2 h at RT. Nuclei were stained with DAPI (Merck, Darmstadt, Germany; #28718-90-3), and a Leica Thunder DMi8 automated microscope was used for image acquisition using a 390 nm UV laser (Leica Microsystems, Wetzlar, Alemania). Image processing was performed with LAS X software.

## Results and Discussion

Three chitosan samples having different molecular weights and a similar DDA were studied for the preparation of fully chitosan-based inks for the 3D printing of films and scaffolds (Table 1). Adhesion and biocompatibility of hRPCs on chitosan films were studied as the first step toward the development of scaffolds suited for the attachment and directed differentiation of hiPSCs to retinal ganglion cells (RGCs).

### Characterization of Chitosan Inks

#### Rheological Properties of Chitosan with Varying *M*_w_ and the Linear Viscoelastic Region

Rheology was used to characterize inks prepared from native chitosan with different *M*_w_’s and a comparable DDA. Strain sweeps were performed for chitosan solutions at constant concentration (6 wt %), from which the linear viscoelastic region (LVR) could be identified by a slight reduction in the loss modulus occurring after 25% strain (Figure 1A). The LVR did not markedly depend on the molecular weight, and all following rheological characterization experiments were therefore performed at a strain below 25%.

**Figure 1:**
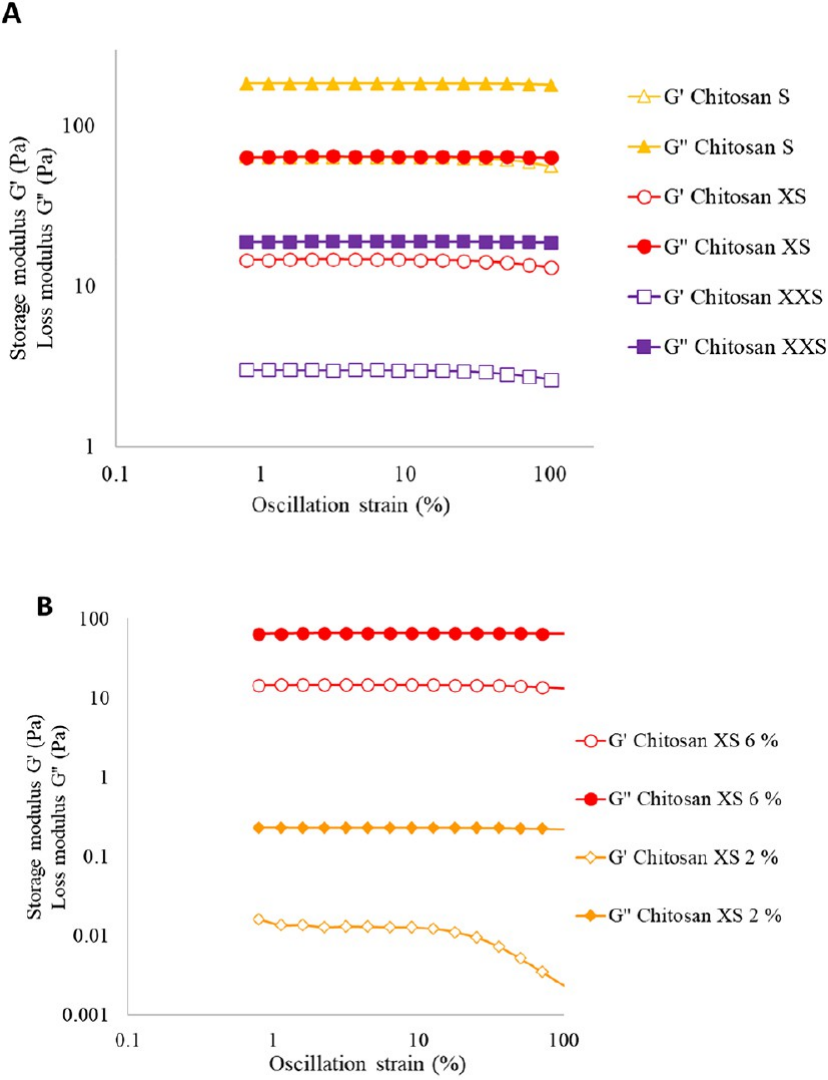
Strain sweep tests (1 Hz, 20 °C) for (A) chitosan XXS, XS, and S (*M*_w_ 99, 201, and 340, respectively) (6 wt %) in AcOH (3%) and (B) chitosan XS at 2 wt % (6 wt % included for comparison, all other parameters kept constant).

The effect of concentration on the LVR was determined by comparing the changes in loss and storage moduli for two chitosan solutions with constant *M*_w_’s at 2 and 6 wt %. At reduced concentrations, the LVR shifted to a strain of 10% (Figure 1B). The greater strength of the samples having a 6 wt % chitosan concentration can likely be attributed to increased chain–chain interactions for higher concentrations, resulting in a stronger network and, hence, a stronger gel structure.

The dynamic rheological properties of the inks were characterized by frequency sweeps performed within the LVR. As previously described by Martínez-Ruvalcaba et al.,^23^ viscous effects dominate the rheological properties of concentrated chitosan solutions (6 wt %). This was evidenced by the loss modulus (*G*″) being greater than the storage modulus (*G*′) across a wide range of frequencies (Figure S1). The crossover shifted to an increased frequency for a decreased *M*_w_, and for chitosan XXS, *G*″ was greater than *G*′ across all frequencies. This trend has previously been observed and attributed to increased relaxation time with increasing *M*_w_.^24^

#### Complex Viscosity—Shear Thinning Properties of Chitosan

Viscosity control is important for the printing of well-resolved architectures. When printing with inks having varying molecular weights, changes in viscosity are oftentimes compensated for by adjusting the concentration. Concentration, in turn, affects both porosity and mechanical properties, both of which are important when preparing scaffolds for biomedical applications. In the case of chitosan, the temperature and rate of deformation also affect the resulting viscosity of a solution. Aiming to identify strategies for 3D printing of varying *M*_w_’s at constant concentration, the effect of temperature and the rate of deformation was determined for chitosan inks prepared from the three *M*_w_’s at constant concentration (6 wt %). The complex viscosity also depends on both the temperature and the rate of deformation, with the effects increasing for increasing *M*_w_’s. Effects were the largest for the high-*M*_w_ samples (340 kDa, chitosan S), for which both the increased rate of deformation (in the range 0.2 to 100 Hz) and increased temperature (10 to 35 °C) (Figure 2) were accompanied by a fivefold reduction in complex viscosity. While a marked reduction in the viscosity of chitosan S (340 kDa) could be observed, only small effects were observed for chitosan XXS (99 kDa) across a range of frequencies and temperatures. While both the temperature and shear rate did affect the viscosity, neither resulted in comparable viscosity for the different *M*_w_’s (effects were not large enough within the accessible range). Printing was therefore continued at ambient conditions (20 °C), under which it could be performed with all three inks. Similar parameters were used for the three inks, except for the extrusion speed and head speed, which were adapted to obtain continuous and uniform printing. However, the viscosity of the gels at room temperature was not high enough to provide good shape fidelity. Therefore, the Regemat3D bioprinter was upgraded with a freezing system (Figure S2). Freezing of the printing bed was performed to maintain the shape of the printed scaffolds. A temperature of −15 °C was used for the bed, while the syringe was maintained at room temperature to ensure good flowability of the inks in the syringe and retention of architecture on the bed for all chitosan types at 6 wt %.

**Figure 2:**
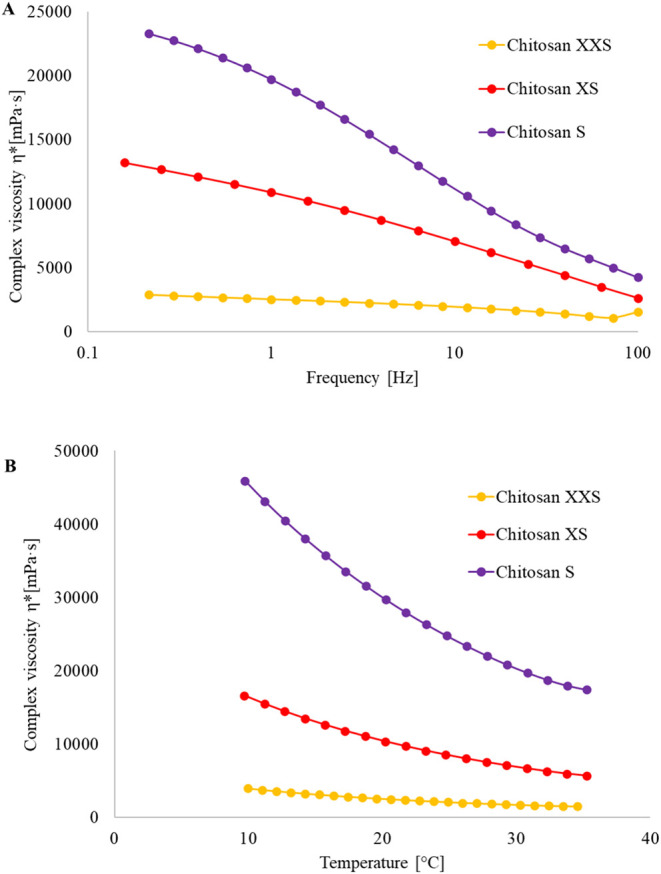
Complex viscosity of chitosan XXS, XS, and S inks (6 wt %) with varying *M*_w_’s (99, 201, and 340 kDa, respectively) as a function of frequency (A) and temperature (B).

#### 3D Printed Scaffolds with a Grid-Type Architecture

Chitosan XS (*M*_w_ 201) was used to optimize the stabilizing strategy, and for this study, several scaffolds were printed with the same printing parameters and frozen. Different stabilizing strategies were used to gel and dry the scaffolds (Table 3). Stabilization was performed by taking advantage of the pH-dependent solubility of chitosan resulting from its polyelectrolyte properties. The printed constructs were immersed in sodium hydroxide (1 M) to revert the amino group to its deprotonated form and thus stabilize the structure. Figure 3 shows 3D printed scaffolds exemplifying print fidelity and scaffold shrinkage after drying for the four different strategies tested in this study (Table 3). Visually, it can be observed that the shape varied depending on the method used for washing and drying (Figure 3). The scaffolds stabilized by strategy 3 (sample EtOH/water/air) deformed, while the one stabilized with strategy 2 (sample EtOH/water/freeze-drying) retained the targeted shape (Figure 3, upper panel). Shrinkage was quantified by image analysis. Shrinkage occurred predominantly during drying, and more shrinkage could be observed in air-dried samples.

**Figure 3:**
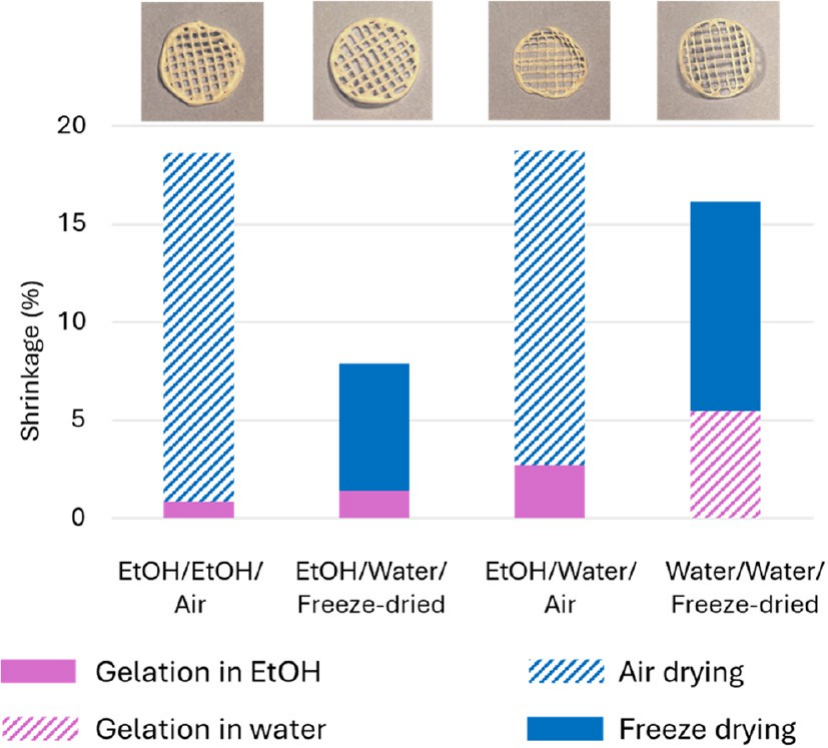
3D printed scaffolds prepared with chitosan XS (201 kDa, 83% DDA), with the conditions used for gelation, washing, and drying indicated. A complete overview is given in Table 3.

For the following experiments regarding 3D printed porous scaffolds, we chose strategy 2, i.e., fixation in ethanol, washing with water, and freeze-drying (EtOH/water/freeze-drying, Table 3) because it led to minimal shrinkage and improved shape fidelity, as demonstrated in Figure 3.

The SEM images show the morphology of 3D printed porous scaffolds (Figure 4). Both the porosity and architecture are key parameters for materials prepared for use as structural scaffolds, enabling functions such as filling void spaces and ensuring cell attachment and differentiation.^25^ However, there is not one optimal porosity and pore structure for all cell lines; the best-suited porosity varies for different cells. Note the apparent increased stability of the architecture of scaffolds prepared by EtOH/water/freeze-drying (sample 2); i.e., more stable thread dimensions (Figure 4, left) and micropore structure (Figure 4, right) can be observed. The pores have dimensions of roughly 30–50 μm, a dimension that may be considered appropriate for cell nutrition, proliferation, and migration, given that the ganglion cells have sizes in the range of 8–20 μm.^26^ It can be noted that the filament fixed in water (number 4) shows high shrinkage compared with the others fixed in ethanol.

**Figure 4:**
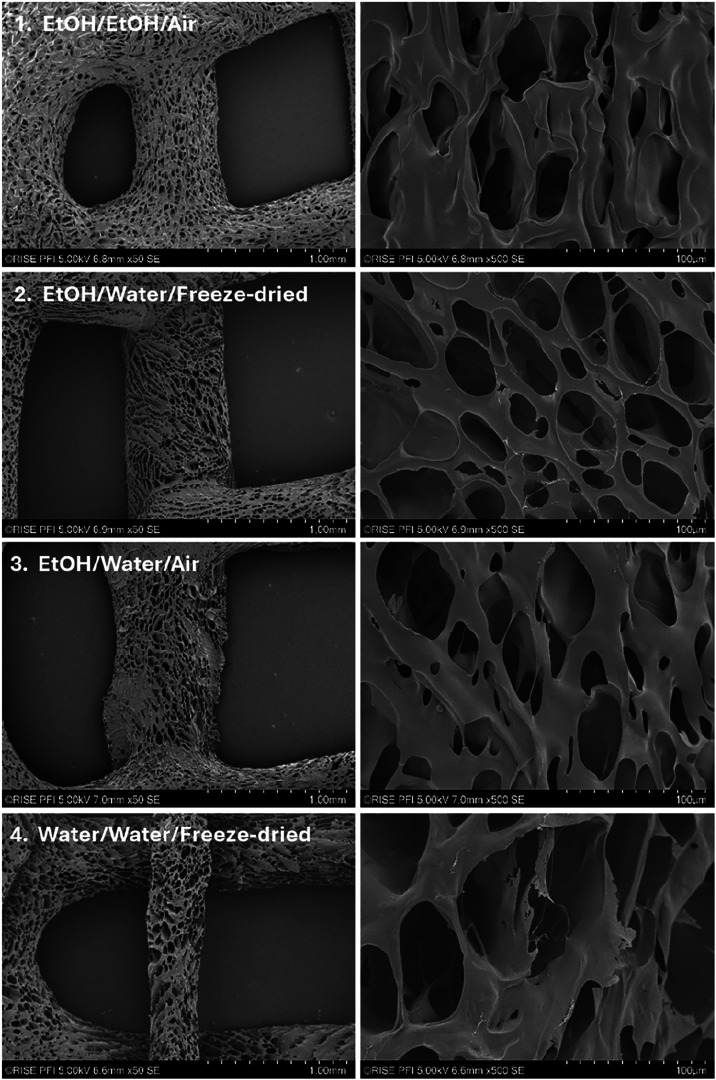
SEM images of 3D printed scaffolds of chitosan XS (201 kDa, 83% DDA) fixated in EtOH/NaOH (−27 °C) (1–3) or 1 M NaOH (4), followed by washing with either ethanol followed by air-drying or by water followed by freeze-drying or air-drying. For details, see Table 3.

#### Surface Functionalization with RGD-Containing Peptides

The molecular weight of chitosan has previously been shown to influence mechanical properties,^27^ which in turn is important for cell adhesion and proliferation. To first evaluate the effect of the chitosan *M*_w_ on hRPCs, we first prepared scaffolds from the three different chitosan *M*_w_’s (at a constant 6 wt % ink concentration). However, preliminary trials showed that none of the three tested chitosan types (*M*_w_ 99–340 kDa) promoted cell adhesion (Figure S3). Hence, it was decided that surface properties should be optimized for improved cell adhesion. Considering that chitosan XS has a *M*_w_ in the range previously reported as best suited for attachment and proliferation for condrocytes,^27^ as well as suitable rheology (Figure 1) and good printability (Figure 3), we focused our efforts on this intermediate-*M*_w_ sample (201 kDa).

Aiming to obtain scaffolds with improved adhesive properties for hRPCs, 3D printed chitosan XS scaffolds (201 kDa, DDA 83%, see Figure 3, upper panel) were modified postprinting with RGDS peptides, taking advantage of the peptide COOH termini and the amino group of chitosan using carbodiimide chemistry. A short peptide was chosen because the RGD motif can be less accessible in high-molecular-weight biopolymers due to chain conformation and steric constraints. Modifications were performed after printing to favor surface substitution and avoid peptides being buried within the scaffold matrix. An unwanted side reaction that may occur under these conditions is self-condensation of the peptide; however, we assume that the high availability of chitosan amino groups (DDA 81–85%) results in the preferential reaction of chitosan amino groups over the amino termini of the peptide. Additionally, Taylor et al.^17^ showed that despite a potential oligomerization of the peptide, all of the chitosan amino groups with a comparable DDA were grafted with peptides or oligomers of peptides.

FT-IR was used to characterize the scaffolds before and after peptide grafting (Figure 5). As indicated in the figure, the intensity of the signal corresponding to the N–H bending of chitosan primary amines at 1580 cm^–1^ decreased after grafting, in good agreement with the formation of amides after the reaction with the COOH termini of the RGD peptide. Notably, the C=O stretching signal at 1650 cm^–1^ from acetylated units remains unchanged, also in good agreement with the selective grafting of chitosan primary amines.

**Figure 5:**
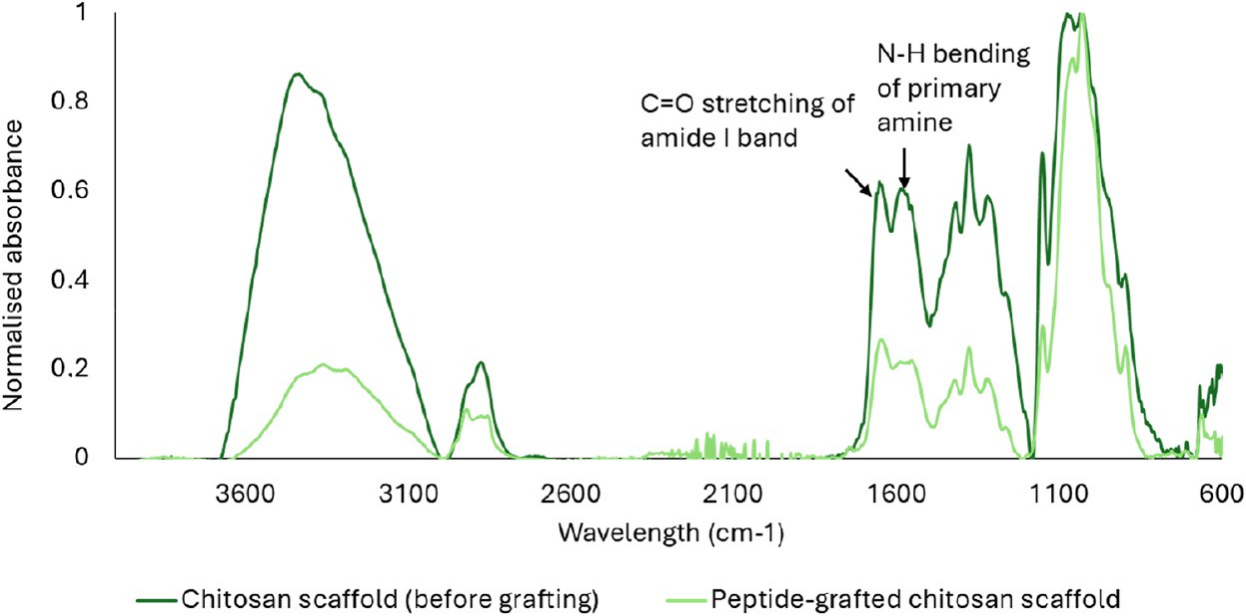
FT-IR spectra of 3D printed chitosan XS scaffolds before and after grafting with RGD peptides. The spectra are normalized using the C–O–C signal at 1029 cm^–1^.

### hRPC Culture on Chitosan

#### hRPC Culture on 2D Chitosan Films

Because of the high opacity of 3D printed scaffolds in the culture media, light microscopy could not be used to study the cell morphology and interaction with the material. 2D films of chitosan were therefore used as models to evaluate the biocompatibility of chitosan with hRPCs and cell adhesion on the material. Four different chitosan 2D films were tested. Films were prepared by air- and freeze-drying to evaluate the effect of drying on hRPC adhesion. Drying is expected to influence the surface characteristics of the films because freeze-drying is expected to largely retain a porous network, while capillary forces will cause collapse of the pores and form a more-dense surface. Air- and freeze-dried films were prepared with and without peptide grafting to determine the effect of an RGD peptide on the adhesion and proliferation of hRPCs. Each experimental condition was tested in three independent replicates, and Matrigel was used as the positive control.

The behavior of hRPCs when seeded on chitosan films and Matrigel was studied by bright-field microscopy. Figure 6 shows representative images of the materials on different days of cell culture. It is possible to distinguish the biomaterial (black arrows), live cells (blue arrows), and dead cells (red arrows). In the Matrigel sample, the hRPCs are visualized as individual cells that grow and replicate over the culture time until day 18. From this moment on, their morphology is worse, and cells appeared abnormally clustered. The hRPCs on the air-dried chitosan film have a different aspect from those on the positive control. It also appears that the cells progressively die and take on a rounded shape.

**Figure 6:**
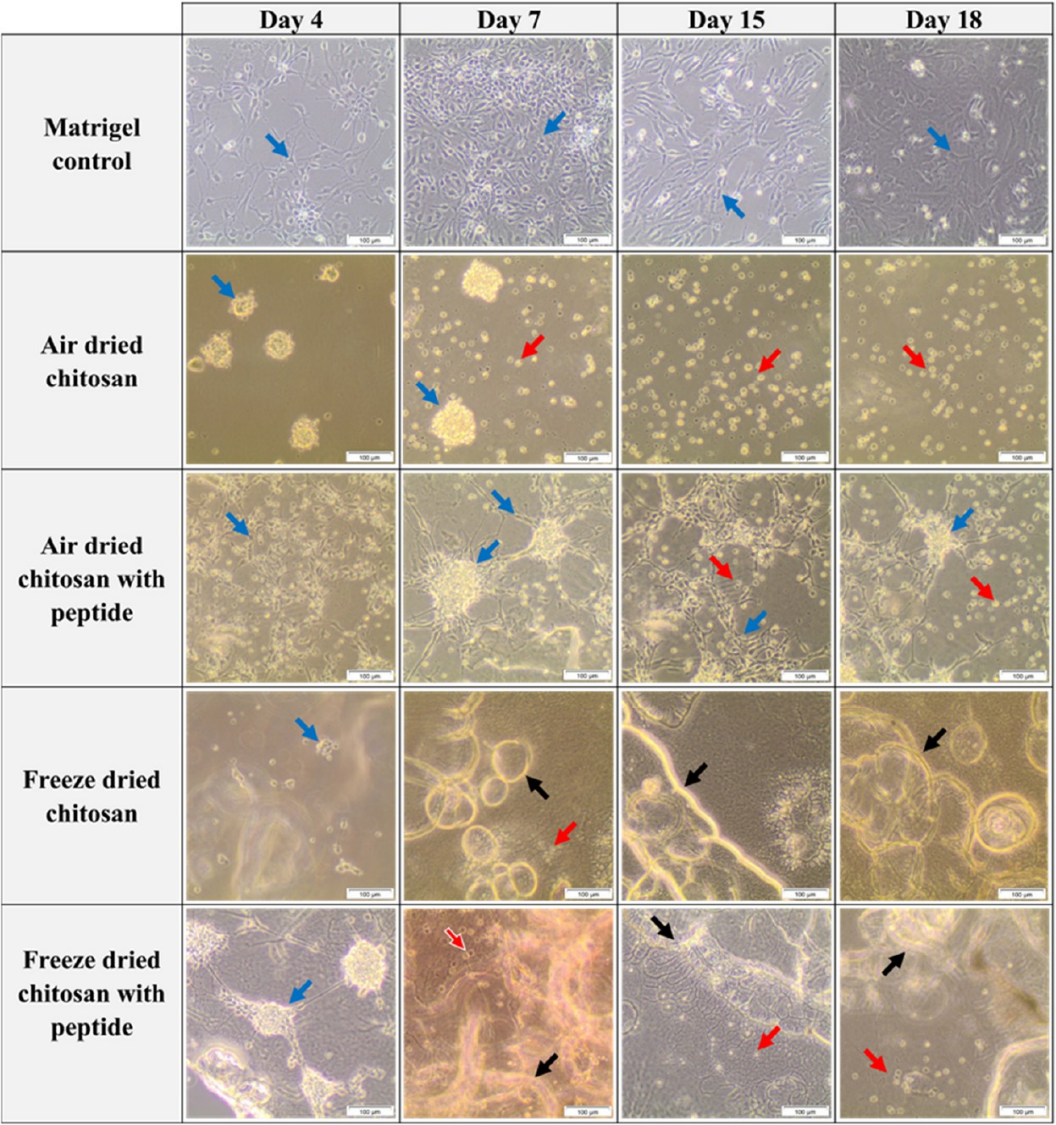
Cell culture results of hRPCs seeded on Matrigel and air- and freeze-dried chitosan films with and without the peptide. Each experimental condition was tested in three independent replicates. Representative images were obtained with bright-field microscopy (10× objective). In this figure, the biomaterial (black arrows), live cells (blue arrows), and dead cells (red arrows) are shown. Matrigel sample, hRPCs are visualized as individual cells; air-dried chitosan film, a few cells with a rounded shape can be seen; air-dried chitosan with the peptide film, the cells have axons and group together forming bigger structures; freeze-dried chitosan film, a few live cells are deposited on the sample; and freeze-dried chitosan film with the peptide, cells group together and progressively detach from the biomaterial. Scale bar: 100 μm.

For the peptide-grafted, air-dried chitosan film, multiple cells can be seen throughout the entire cell culture period. Remarkably, these cells present axons and group together forming bigger structures similar to those obtained in previous studies.^28,29^ Chen et al. showed that hRPCs from mouse (mRPCs) seeded on cationic chitosan-*graft*-poly­(ε-caprolactone)/polycaprolactone hybrid scaffolds prepared by electrospinning adopted a spherical morphology and neuron-like morphology with bipolar or multipolar extensions.^28^ In addition, Jiang et al. showed that mRPCs seeded on a chitosan hydrochloride and oxidized dextran hydrogel tended to expand with larger cellular clusters and higher cell densities than in the positive controls.^29^

For the freeze-dried chitosan film, a few live cells are deposited on the sample (Figure 6). However, due to the structure of the biomaterial, it is not possible to distinguish anything except the chitosan film. For the peptide-grafted freeze-dried film, cells very similar to those observed for the peptide-grafted air-dried film can be observed initially. However, with increased time, a detachment can be observed. The difference may result from the difference in the higher surface roughness and porosity of the freeze-dried films compared to the air-dried films (Figure 7).

**Figure 7:**
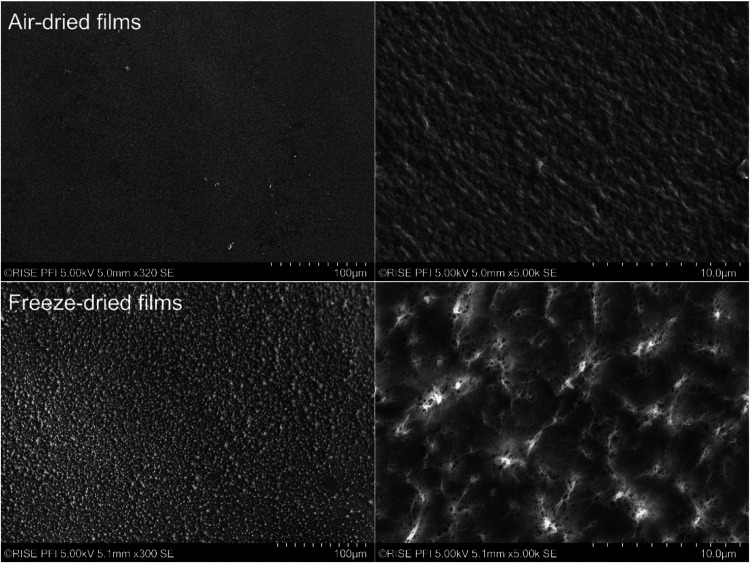
Surface SEM images of chitosan XS films prepared by air-drying (upper panel) and freeze-drying (lower panel). Note the rougher surface of the freeze-dried films (lower panel), which is an indication of higher surface porosity.

The results show that chitosan is a biocompatible support material for the hRPCs, and the peptide-grafted air-dried chitosan films are best suited for promoting adhesion of hRPCs. The results agree with those reported by Ho et al.;^15^ they showed that chitosan scaffold functionalization with a peptide including the RGD sequence enhanced attachment and proliferation of rat osteosarcoma cells. In addition, recent studies also confirmed the important role of the RGD sequence by performing similar experiments to those described in the present article, employing human dental pulp stem cells seeded on chitosan scaffolds, ref 16. The improved cell adhesion observed for RGD-grafted scaffolds is in line with the literature for RGD-grafted biomaterials.^30,31^ We hypothesize that the more collapsed surface and reduced porosity of the air-dried films favor peptide substitution on the surface. Surface availability will likely improve cell adhesion and contribute to the improved cell response observed for this material. Limited information is available on the preference of hRPCs with regard to material properties like stiffness and porosity. Although the results seem to indicate that the cells prefer a film-type surface, further studies are needed to understand the relationship between material properties and the adhesion and proliferation of hRPCs.

When looking at the morphology of the hRPCs seeded on the RGD-grafted chitosan film, it seems that the cells could be differentiating toward another retinal cell type. A similar phenomenon has been demonstrated by Chen et al., as they observed that nanofibrous scaffolds favored the proliferation and differentiation of mRPCs toward the neuronal lineage.^28^ However, additional studies are needed to further understand the state of differentiation of the cells grown on chitosan films.

#### hRPC Culture on 3D Printed Chitosan Scaffolds

Once the biocompatibility of the 2D chitosan films with hRPCs was confirmed, further experiments with 3D printed porous scaffolds were performed to study their potential with hRPCs. Like the chitosan films, a bright-field microscope was used to try to analyze the hRPC behavior; however, only the scaffold structure was observed using this type of microscopy due to the high opacity of the scaffolds. The presence of cells deposited on chitosan scaffolds could be confirmed by DAPI staining analyses, which showed the presence of numerous marked nuclei (Figure 8). Moreover, it appears that the chitosan 3D printed scaffold modified with the peptide, including the RGD sequence, presented a greater number of cells than the 3D printed chitosan scaffold without modification. Although DAPI staining primarily labels nuclei regardless of cell viability, this analysis appears to be in line with the results obtained for chitosan 2D films; i.e., the RGD sequence seems to improve the adhesion of hRPCs to chitosan scaffolds. However, further studies are needed to characterize the hRPCs on RGD-modified 3D printed scaffolds with the final goal of using these biomaterials for stem-cell-based therapy for patients with retinal degenerative diseases.

**Figure 8:**
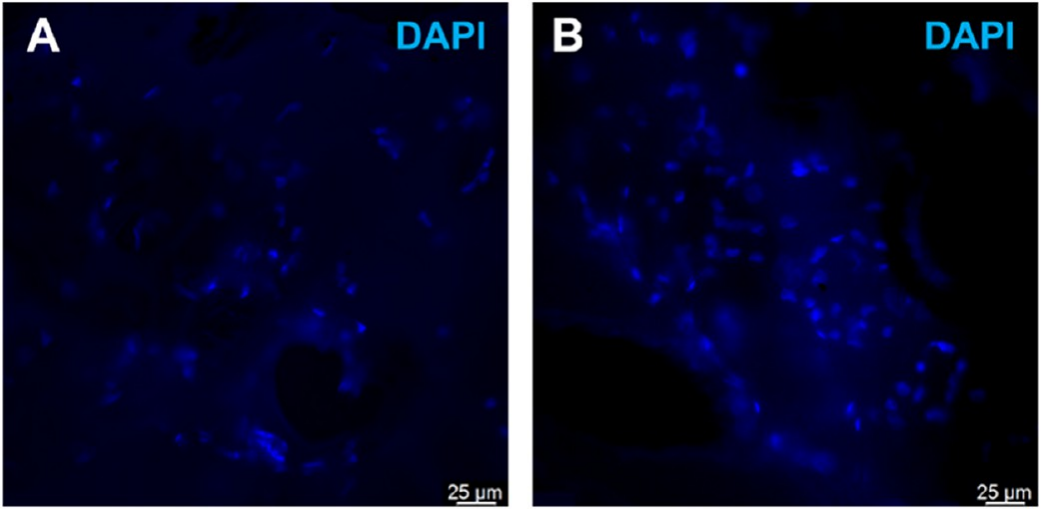
DAPI staining analysis to evaluate the presence of hRPCs on the scaffolds at day 6 of cell culture. Representative confocal images with a 40x objective show the nuclei counterstained with DAPI. (A) Chitosan XS 3D printed scaffold. (B) Chitosan XS 3D printed scaffold modified with peptides that contain the RGD sequence. Scale bars: 25 μm.

## Conclusions

Taken together, films and well-defined scaffolds could be prepared by 3D printing of chitosan inks. Three different *M*_w_’s were studied, and rheological characterization revealed marked differences in the complex viscosity for the three *M*_w_’s studied, i.e., 340, 201, and 99 kDa. By tuning the 3D printing parameters, scaffolds with well-defined architectures could be printed for all three molecular weights. Additionally, fixation and drying were optimized, and shrinkage could be significantly reduced by stabilizing the scaffolds in ethanol, followed by freeze-drying. Scaffolds and films were prepared to study the adhesion and proliferation of hRPCs on native chitosan as a first step toward exploring the suitability of chitosan as a support material for the directed differentiation of hiPSC to RGCs. While the cells did not adhere to native unmodified chitosan, RGD-modified chitosan showed significantly improved adhesion of hRPCs. Moreover, differences could be observed between the scaffolds and films, which, as a direct consequence of their preparation, had significantly different surface morphology. For the chitosan films with the peptide, it seems that the cells could be differentiating toward another retinal cell type, aided by the biomaterial. The results add to the data available for the effect of the biomaterial on hRPCs, and with further studies characterizing the hRPCs on scaffolds, the knowledge may aid in the development of stem-cell-based therapy for patients with retinal degenerative diseases. In conclusion, the work demonstrates the printability of native chitosan inks for the preparation of scaffolds suitable as structural components for seeding of hRPCs, and the detailed chemistry of the scaffolds can be tuned by postprint grafting with relevant biomolecules by taking advantage of chitosan amino groups.

## Supplementary Material



## Data Availability

The data underlying this study are available in the published article and its Supporting Information.

## Notes

Rheological assessment of chitosan samples XXS, XS, and S at 6% concentration, dissolved in 3% AcOH (Figure S1); picture showing the Regemat3D printing setup with a refrigerator system (Figure S2); light microscopy used to assess the effect of chitosan *M_w_* on hRPC culture (Figure S3) (PDF)

## References

[1] Bovi
dos Santos G., de Lima-Vasconcellos T. H., Móvio M. I., Birbrair A., Del Debbio C. B., Kihara A. H. (2024). New Perspectives
in Stem Cell Transplantation and Associated Therapies to Treat Retinal
Diseases: From Gene Editing to 3D Bioprinting. Stem Cell Rev. Rep..

[2] Ortuño-Costela M. d. C., Cerrada V., García-López M., Gallardo M. E. (2019). The challenge
of bringing iPSCs to the patient. Int. J. Mol.
Sci..

[3] Murphy A. R., Haynes J. M., Laslett A. L., Cameron N. R., O’Brien C. M. (2020). Three-dimensional
differentiation of human pluripotent stem cell-derived neural precursor
cells using tailored porous polymer scaffolds. Acta Biomater..

[4] Liu J., Sun L., Xu W., Wang Q., Yu S., Sun J. (2019). Current advances
and future perspectives of 3D printing natural-derived biopolymers. Carbohydr. Polym..

[5] Zhao T., Terracciano R., Becker J., Monaco A., Yilmaz G., Becer C. R. (2022). Hierarchy of complex Glycomacromolecules:
from controlled
topologies to biomedical applications. Biomacromolecules.

[6] Brockhausen I., Schutzbach J., Kuhns W. (1998). Glycoproteins and their relationship
to human disease. Cells Tissues Organs.

[7] Rinaudo M. (2006). Chitin and
chitosan: Properties and applications. Prog.
Polym. Sci..

[8] Vårum K. M., Ottøy M. H., Smidsrød O. (1994). Water-solubility of partially N-acetylated
chitosans as a function of pH: effect of chemical composition and
depolymerisation. Carbohydr. Polym..

[9] Villar-Chavero M. M., Domínguez J. C., Alonso M. V., Oliet M., Rodriguez F. (2019). Tuning the
rheological properties of cellulosic ionogels reinforced with chitosan:
The role of the deacetylation degree. Carbohydr.
Polym..

[10] Lieder R., Darai M., Thor M. B., Ng C. H., Einarsson J. M., Gudmundsson S., Helgason B., Gaware V. S., Másson M., Gíslason J. (2012). In vitro bioactivity of different degree
of deacetylation chitosan, a potential coating material for titanium
implants. J. Biomed. Mater. Res., Part A.

[11] Srivastava G. K., Rodriguez-Crespo D., Singh A. K., Casado-Coterillo C., Fernandez-Bueno I., Garcia-Gutierrez M. T., Coronas J., Pastor J. C. (2014). Chitosan
feasibility to retain retinal stem cell phenotype and slow proliferation
for retinal transplantation. BioMed Res. Int..

[12] Rajabi M., McConnell M., Cabral J., Ali M. A. (2021). Chitosan hydrogels
in 3D printing for biomedical applications. Carbohydr. Polym..

[13] Lawrence B. J., Madihally S. V. (2008). Cell colonization in degradable 3D
porous matrices. Cell Adhes. Migr..

[14] Hansson A., Hashom N., Falson F., Rousselle P., Jordan O., Borchard G. (2012). In vitro evaluation
of an RGD-functionalized
chitosan derivative for enhanced cell adhesion. Carbohydr. Polym..

[15] Ho M.-H., Wang D.-M., Hsieh H.-J., Liu H.-C., Hsien T.-Y., Lai J.-Y., Hou L.-T. (2005). Preparation
and characterization
of RGD-immobilized chitosan scaffolds. Biomaterials.

[16] Asghari
Sana F., Çapkın Yurtsever M., Kaynak Bayrak G., Tunçay E. Ö., Kiremitçi A. S., Gümüşderelioğlu M. (2017). Spreading, proliferation and differentiation
of human dental pulp stem cells on chitosan scaffolds immobilized
with RGD or fibronectin. Cytotechnology.

[17] Taylor D. L., Thevarajah J. J., Narayan D. K., Murphy P., Mangala M. M., Lim S., Wuhrer R., Lefay C., O’Connor M. D., Gaborieau M., Castignolles P. (2015). Real-time monitoring of peptide grafting
onto chitosan films using capillary electrophoresis. Anal. Bioanal. Chem..

[18] Chinga-Carrasco G., Ehman N. V., Filgueira D., Johansson J., Vallejos M. E., Felissia F. E., Håkansson J., Area M. C. (2019). Bagasse—A major agro-industrial residue as potential
resource for nanocellulose inks for 3D printing of wound dressing
devices. Addit. Manuf..

[19] Rogina A., Pribolšan L., Hanžek A., Gómez-Estrada L., Ferrer G. G., Marijanović I., Ivanković M., Ivanković H. (2016). Macroporous
poly (lactic acid) construct supporting
the osteoinductive porous chitosan-based hydrogel for bone tissue
engineering. Polymer.

[20] Galera-Monge T., Zurita-Díaz F., Canals I., Grønning
Hansen M., Rufián-Vázquez L., Ehinger J. K., Elmér E., Martin M. A., Garesse R., Ahlenius H., Gallardo M. E. (2020). Mitochondrial
dysfunction and calcium dysregulation in leigh syndrome induced pluripotent
stem cell derived neurons. Int. J. Mol. Sci..

[21] Lee J., Choi S.-H., Kim Y.-B., Jun I., Sung J. J., Lee D. R., Kim Y. I., Cho M. S., Byeon S. H., Kim D.-S., Kim D. W. (2018). Defined conditions for differentiation
of functional retinal ganglion cells from human pluripotent stem cells. Invest. Ophthalmol. Visual Sci..

[22] García-López M., Jiménez-Vicente L., González-Jabardo R., Dorado H., Gómez-Manjón I., Martín M. Á., Ayuso C., Arenas J., Gallardo M. E. (2024). Creation of an Isogenic
Human iPSC-Based RGC Model of Dominant Optic Atrophy Harboring the
Pathogenic Variant c. 1861C> T (p. Gln621Ter) in the OPA1 Gene. Int. J. Mol. Sci..

[23] Martínez-Ruvalcaba A., Chornet E., Rodrigue D. (2004). Dynamic Rheological Properties of
Concentrated Chitosan Soltions. Appl. Rheol..

[24] Hou Z., Zhang M., Liu B., Yan Q., Yuan F., Xu D., Gao Y. (2012). Effect of
chitosan molecular weight on the stability
and rheological properties of β-carotene emulsions stabilized
by soybean soluble polysaccharides. Food Hydrocolloids.

[25] Stella J. A., D’Amore A., Wagner W. R., Sacks M. S. (2010). On the biomechanical
function of scaffolds for engineering load-bearing soft tissues. Acta Biomater..

[26] Hebel R., Holländer H. (1983). Size and distribution
of ganglion cells in the human
retina. Anat. Embryol..

[27] Hsu S.-h., Whu S. W., Tsai C.-L., Wu Y.-H., Chen H.-W., Hsieh K.-H. (2004). Chitosan as scaffold
materials: Effects of molecular
weight and degree of deacetylation. J. Polym.
Res..

[28] Chen H., Fan X., Xia J., Chen P., Zhou X., Huang J., Yu J., Gu P. (2011). Electrospun chitosan-graft-poly­(ε-caprolactone)/poly­(ε-caprolactone)
nanofibrous scaffolds for retinal tissue engineering. Int. J. Nanomed..

[29] Jiang F., Tang Z., Zhang Y., Ju Y., Gao H., Sun N., Liu F., Gu P., Zhang W. (2019). Enhanced proliferation
and differentiation of retinal progenitor cells through a self-healing
injectable hydrogel. Biomater. Sci..

[30] Alsberg E., Anderson K. W., Albeiruti A., Rowley J. A., Mooney D. J. (2002). Engineering
growing tissues. Proc. Natl. Acad. Sci. U.S.A..

[31] Place E. S., Evans N. D., Stevens M. M. (2009). Complexity in biomaterials for tissue
engineering. Nat. Mater..

