# Supplementary material for: 3D Printing of Chitosan Scaffolds and Films with Varying Roughness for Cultivation of Human Retinal Progenitor Cells
Source: ACS Omega. 2025 Sep 26;10(39):45342–52. doi: 10.1021/acsomega.5c05018 (PMC12508935; doi:10.1021/acsomega.5c05018)
Supplement: Supplementary file 1 [file ao5c05018_si_001.pdf]

Supporting information

3D printing of chitosan scaffolds and films with varying roughness for cultivation of human retinal progenitor cells

*Amalie Solberg1, Natalia Robles-Anda2, Eva Pasquier1, Helena Dorado Monreal2, Miguel Ladero3, M. Esther Gallardo2, * and Gary Chinga-Carrasco1,**

[1] RISE PFI, Høgskoleringen 6b, Trondheim, Norway

[2] Translational Research with iPS Cells Group, Research Institute of Hospital 12 de Octubre, imas12, 28041 Madrid, Spain

[3] FQPIMA group. Chemical Engineering and Materials Department, Universidad Complutense de Madrid. 28040 Madrid, Spain

[*] Corresponding authors: gary.chinga.carrasco@rise-pfi.no and egallardo.imas12@h12o.es

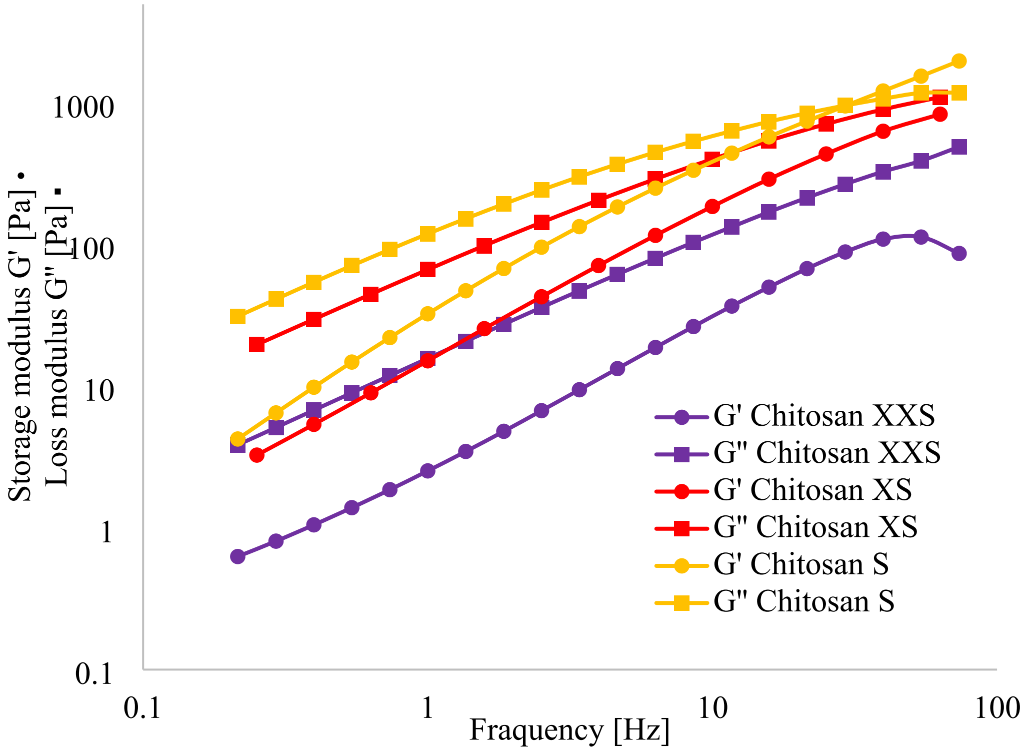

Figure S1. Frequency sweep (10 % strain and 20 °C) performed for chitosan samples (Chitosan XXS, XS and S, 6 % conc.) with varying MW (respectively, 99, 201 and 340 kDa) and similar DDA (81 – 85 %). All samples were dissolved in 3 % AcOH.

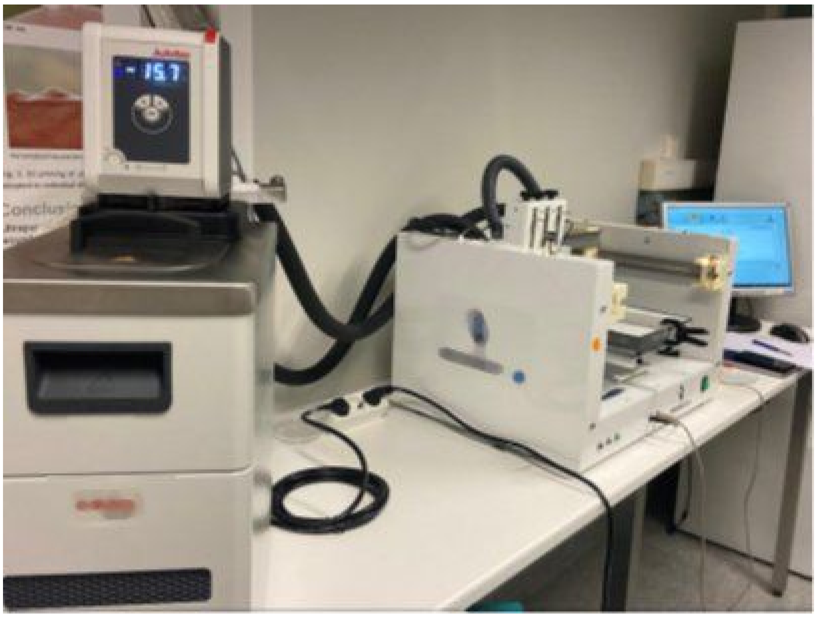

Figure S2. Regemat3D printing set-up with a refrigerator system.

To first evaluate the effect of the chitosan MW on hRPCs culture, scaffolds were prepared from chitosan with three different MWs (at a constant 6 wt.% ink concentration). Then, $0.45 \cdot 10^6$ hRPCs in 750 µL of N2B27 medium were seeded into these samples and incubated at 37 ºC and 5% CO_2. hRPCs were cultured on the materials for ten days by changing the culture medium every day. Light microscopy was used to follow the process and at the end of the culture period a representative image was acquired using an Olympus SC30 camera (Figure S3).

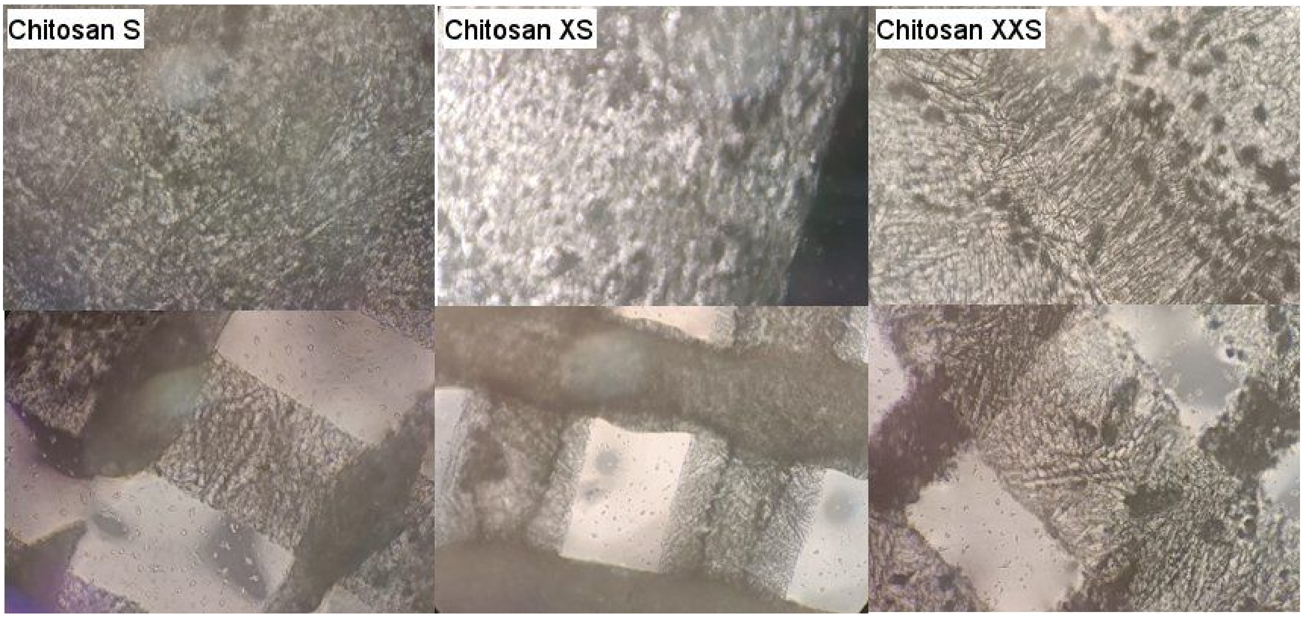

Figure S3. Light microscopy images of scaffolds with hRPCs after 10 days of cell culture, in parallel (upper panel) and perpendicular geometry (lower panel).
